# Sexual pain and IC/BPS in women

**DOI:** 10.1186/s12894-019-0478-0

**Published:** 2019-06-06

**Authors:** Su Jin Kim, Jayoung Kim, Hana Yoon

**Affiliations:** 1 Department of Urology, Seoul St. Mary’s Hospital, The Catholic University of Korea College of Medicine, Seoul, Republic of Korea; 2 Departments of Surgery and Biomedical Sciences, Cedars-Sinai Medical Center, Los Angeles, CA 90048 USA; 3 Department of Medicine, University of California Los Angeles, Los Angeles, CA 90095 USA; 4 Department of Urology, Medical Research Center, Ewha Womans University School of Medicine, Seoul, Republic of Korea; 5 Current address: Department of Urology, Yonsei University Wonju College of Medicine, Wonju, Republic of Korea

**Keywords:** Cystitis, interstitial, Sexual dysfunction, Chronic pain, Vulvodynia, Lower urinary tract symptoms, Quality of life

## Abstract

Interstitial cystitis/bladder pain syndrome (IC/BPS) and female sexual dysfunction (FSD) are common conditions that substantially reduce women’s health. In particular, women with IC/BPS show vulvodynia, a kind of FDS that originates from consistent pain around the vulvar area. There have been many studies attempting to find the underlying mechanisms that induce the chronic pain associated with IC/BPS and vulvodynia and explain why these two conditions often coexist. Proposed theories suggest that pain hypersensitivity is being mediated by peripheral and central sensitization. However, there are still many unknown factors, such as etiologies, that can evoke pain hypersensitivity and may be linking the casual relationship between IC/BPS and vulvodynia. At present, knowledge regarding IC/BPS and vulvodynia are insufficient when considering their clinical importance. Therefore, efforts are necessary to elucidate the issues surrounding IC/BPS and vulvodynia.

## Background

Female sexual dysfunction (FSD) and interstitial cystitis/bladder pain syndrome (IC/BPS) are two conditions affecting women’s health. A study on the association between sexual and general well-being found that women reported better quality of life (QoL) with higher sexual satisfaction, regardless of age and/or menopausal status [1]. Both FSD and IC/BPS significantly impairs a woman’s abilities to pursue and enjoy sexual relations. Approximately 40–50% of women experience FSD and 0.5–12% experience IC/BPS. Considering these incidence rates, both FSD and IC/BPS present serious challenges for patients and clinicians [2–6].

Chronic pain deteriorates not only personal health and wellness, but also QoL. Chronic pain can induce sexual dysfunction, such as arousal disorder, and relationship problems [7]. Furthermore, studies have shown that significantly more women with chronic pelvic pain (CPP) show FSD compared to those without CPP. Women with CPP and FSD reported various types of sexual dysfunctions, including hypoactive sexual desire disorder, sexual arousal disorder, orgasmic disorder, and sexual pain disorder [8].

The symptoms of IC/BPS, such as urinary frequency, urgency, and pelvic pain, can have a negative impact on sexual activity and QoL [9]. Women with overactive bladder (OAB) frequently have a risk for sexual dysfunction [10]. In the postmenopausal group, women with scores indicating severe OAB reported worse sexual function, particularly in the arousal, lubrication, orgasm, pain, and total domains [11]. Despite the known association between FSD and bladder diseases, contributing risk factors have yet to be explored. Evaluating the impact of duration, severity, pain localization, sexual trauma history, anxiety, and depression associated with sexual dysfunction may help elucidate risk factors. Increasing our knowledge about sexual dysfunction as it relates to bladder diseases may aid in clinical diagnoses, treatment strategies, and overall symptom improvement. This review will provide an overview of studies that address FSD in women with IC/BPS.

### Pain in FSD

According to consensus from the 4th Internaional Consultation on Sexual Medicine (ICSM), FSD is classified as hypoactive sexual desire dysfunction, female sexual arousal dysfunction, female orgasmic dysfunction, female-genital-pelvic pain dysfunction, persistent genital arousal disorder, postcoital syndrome, hypohedonic orgasm, or painful orgasm [2, 12]. Guidelines from the 4th ICSM also define pain-associated FSD as female-genital-pelvic pain dysfunction and include all conditions that inhibit sexual intercourse or induce negative effects on sexual functions. Female-genital-pelvic pain dysfunction is different from the previous classification characterized as pain associated with FSD, which includes sexual pain disorder including dyspareunia and vaginismus [13]. According to the ICSM, female-genital-pelvic pain dysfunction includes persistent or recurrent difficulties with at least one of the following: (1) vaginal penetration during intercourse, (2) marked vulvovaginal or pelvic pain during genital contact, (3) marked fear or anxiety about vulvovaginal or pelvic pain in anticipation of, during, or as a result of genital contact, or (4) marked hypertonicity or overactivity of pelvic floor muscles with or without genital contact [14]. Painful orgasm also falls under this category and is defined as genital and/or pelvic pain during or shortly after orgasm [2]. These recent new definitions reflect the evolving concept behind pain-associated FSD as it also now includes pain in the vulvovaginal and pelvic area and pelvic floor hypertonicity. The new categorization of female-genital-pelvic pain dysfunction has broadened the view on pain-associated FSD and has differentiated it from its previous classification, which confined symptoms to each organ or single disease. This recent change in classification by the ICSM considers pain-associated FDS as a complex condition influenced by psychological and physical factors and supports the general thought that FSD is multifactorial.

Previously, vulvodynia had been generalized under chronic vulvar pain with no precise discrimination for it. However, recent consensus has redefined chronic vulvar pain as vulvar pain associated with specific diseases, such as inflammation, neoplasm, and/or injury. This was conducted at a conference with the International Society for the Study of Vulvovaginal Disease, the International Society for the Study of Women’s Sexual Health, and the International Pelvic Pain Society [15]. Vulvodynia can have diverse pain characteristics; therefore, pain-based classification helps in identification, diagnosis, and treatment. Vulvodynia can be characterized as either general (entire vulva) or localized (parts of the vulva). Additionally, based on the situation of the pain, vulvodynia can be classified as either provoked (triggered by physical contact) or unprovoked (spontaneous occurrence without specific triggers) [16]. Women of all ages can experience vulvodynia, and provoked vulvodynia is the most commonly diagnosed [17, 18]. Provoked vulvodynia is thought to be more widely diagnosed than unprovoked because its symptoms can be better recognized by doctors. Therefore, there is an great need to better identify unprovoked vulvodynia patients.

### Sexual pain and IC/bps

IC/BPS is a disorder that induces chronic pain or discomfort in the bladder and surrounding pelvic organs [19, 20]. At present, IC/BPS is not a disease confined to just the bladder and pelvic area; it is a complex disease that includes the outside of the genitourinary tract. Tripp et al. [21] investigated the pain characteristics of IC/BPS using whole-body diagram pain locators. They found that women with IC/BPS reported significantly more pain all over their body, compared to healthy women without. Only 52 of the 193 IC/BPS diagnosed women (27%) presented pain restricted to the bladder and pelvic area. Moreover, IC/BPS patients had various co-morbidities. Diseases, such as irritable bowel syndrome, fibromyalgia, vulvodynia, chronic pelvic pain, endometriosis, OAB, allergies and chronic fatigue syndrome, were found to coexist in IC/BPS patients [22–28].

In addition, there are several reports that IC/BPS can increase the risk for or worsen other diseases, including FSD. A population-based study found higher prevalence of FSD in women with IC/BPS [29]. FSD incidence also increased depending on the severity of IC/BPS symptoms, suggesting that FSD is a factor may be worsening IC/BPS. In regards to the pain associated with FSD, vulvodynia may be contributing to flare-ups of IC/BPS symptoms and could be the reason why IC/BPS patients avoid sexual activity [9, 30].

### Can FSD and IC/bps be generalized as one disease?

Diseases characterized with lower urinary tract symptoms (LUTS) in females, such as incontinence and OAB, are known to have a negative impact on all domains in female sexual function. Symptoms of IC/BPS can also deteriorate patient’s sexual activities and QoL. A significant number of IC/BPS patients avoid sexual activity because of pain. In addition, FSD and IC/BPS share similar clinical characteristics and comorbidities, making it difficult to discriminate between the two [31–33]. As mentioned previously, there are many clinical reports presenting an association between IC/BPS and vulvodynia, mainly due to shared FSD conditions.

**Fig. 1:**
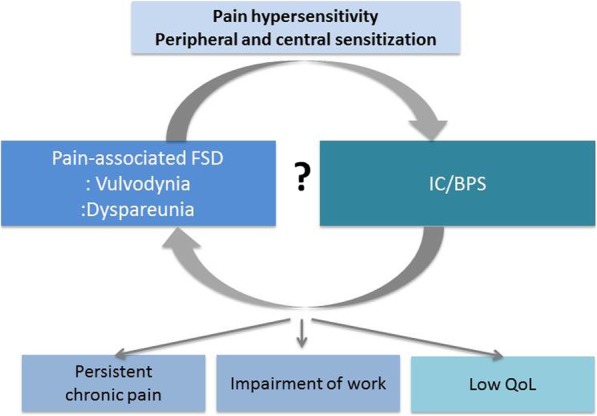
FSD and IC/BPS: FSD; Female sexual dysfunction, *IC/BPS* Interstitial cystitis/bladder pain syndrome, *QoL* Quality of life

Although the mechanisms of the association between IC/BPS and vulvodynia are unclear, visceral nerve cross-talking and the anatomic relationship between genital organs and the bladder offer a simple proximity explanation. Another possible mechanism behind the relationship is abnormal pain hypersensitivity induced by peripheral and central sensitization. The abnormal pain response frequently observed in vulvodynia patients is caused by central or peripheral maladaptive pain processing from local insult, injury, or trauma (Fig. 1).

### Role of peripheral sensitization in the pain hypersensitivity of vulvodynia and IC/BPS

Exposure of nociceptors to repetitive pain stimulation reduces the pain threshold and amplifies the responsiveness of nociceptors. Therefore, this can abnormally increase peripheral transduction of sensitivity and lead to the development of peripheral pain hypersensitivity [34–36]. The potential underlying mechanism of peripheral pain hypersensitivity noted in both vulvodynia and IC/BPS could be due to sensory nerve upregulation. Previous studies have shown that sensory nerve density is significantly increased in the vulva vestibule and bladder. Compared to the normal controls, patients with vulvodynia were found to have increased nociceptors in their vulvar vestibule [37–40]. Consequentially, this increased density of peripheral nociceptors results in increased sensitivity. It was also found that transient receptor potential V1 (TRPV1) exists in these nociceptive nerve endings and enhances pain signaling [37, 41, 42]. Pukall CF et al. [43] and Giesecke J et al. [44] reported increased peripheral tactile, pressure, and pain sensitivity in the patients with vulvodynia and confirmed histologic and molecular changes of peripheral nociceptors is associated with clinical manifestations. Similarly with vulvodynia, it has been reported that IC/BPS patient bladders have upregulated sensory innervation and TRPV1 expression [45–47].

### Role of central sensitization in the pain hypersensitivity of vulvodynia and IC/BPS

Central sensitization is an important mechanism underlying various conditions associated with chronic pain and induces pain hypersensitivity through pathologically enhanced pathways that are not normally associated with nociception. For example, the low-threshold Aδ fiber, that is mostly used for temperature and pressure signaling, can be sensitized to pain. Chronic pain induced by central sensitization is persistent even after initiation of the signal and disappearance of the peripheral cause [48]. Studies have shown that the pain characteristics of central sensitization can be found in vulvodynia patients. Foster DC et al. [49] observed that vulvar vestibulitis syndrome patients experienced hyperalgesia and allodynia more often than normal controls after intradermal foot and forearm capsaicin injections. In addition, pelvic organ crosstalk has an important role in central sensitization because pelvic organs, such as the bladder, colon and vulva, are controlled by the same neural pathway [50]. Thus, afferent signals from other pelvic organs can provoke pain through neural crosstalk even though the initiation and peripheral causes of vulvodynia and IC/BPS have gone. Therefore, central sensitization plays an important role in the chronic pain observed in vulvodynia and IC/BPS. Clinically, similar manifestations of vulvodynia and IC/BPS have also existed. Moreover, recently, there has been an attempt to categorize various pain-associated conditions due to central sensitization as central sensitivity syndrome (CSS). Vulvodynia and IC/BPS are considered subgroups of CSS [51]. Previous studies on clinical findings support the notion that the same mechanisms associated with central sensitization are involved in the pain behind vulvodynia and IC/BPS [52–54].

**Fig. 2:**
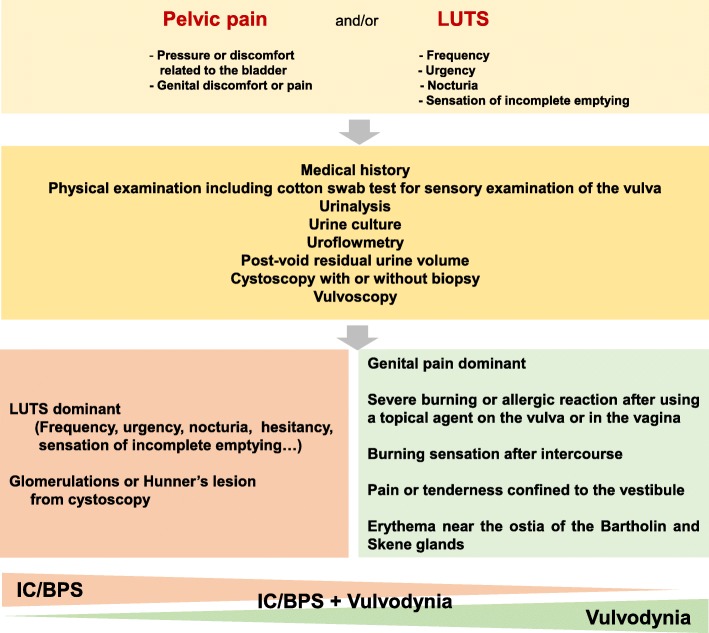
Approach for differential diagnosis of IC/BPS, vulvodynia, and IC/BPS + vulvodynia (*LUTS* lower urinary tract symptoms, *IC/BPS* interstitial cystitis/bladder pain syndrome)

Recently, evidence supporting central sensitization using functional and structural brain imaging were reported in vulvodynia and IC/BPS. Previously, Pukall et al. [55] showed that increased perception and activation of pain-related brain regions were observed in women with vulvar vestivulitis syndrome, compared to normal women, after tacticle stimulation of the vulvar vestibule. Other studies have reported that vulvodynia patients show increased grey matter density in pain-modulating and stress-related regions of the brain as well as alterations in the intrinsic connectivity of regions comprising the sensorimotor, salience, and default mode resting state networks [56, 57]. Similarly, women with IC/BPS showed alterations of oscillation frequency and functional connectivity of brain regions previously reported in other chronic pain conditions [58] and various white matter (right anterior thalamic radiation, left forceps major, and right longitudinal fasciculus, right superior and bilateral inferior longitudinal fasciculi) abnormalities that correlated with severity of pain, urinary symptoms, and impaired QoL [59]. Fig. 2 shows common therapeutic approaches and points of divergence among IC/BPS, IC/BPS + vulvodynia, and vulvodynia patient groups.

### Approach and management of vulvodynia and IC/bps patients

Clinically, vulvodynia and IC/BPS often coexists and differentiation between the two is not easy, especially when the patient reports LUTS combined with pain. A majority of patients also seek medical care after relatively longer periods of initially feeling pain, leading to central sensitization that has been firmly established. Furthermore, clinical manifestations of chronic pain found in various other comorbidities can mask vulvodynia and IC/BPS, making it confusing and difficult to officially diagnose.

Many previous studies have revealed a close correlation between LUTS and impaired sexual function; IC/BPS is not an exception. Besides, it is not uncommon to have trouble differentiating sexual pain and IC/BPS-related genital pain in women. Unfortunately, despite the growing population of affected individuals, there is a lack of comprehensive studies regarding sexual dysfunction and IC/BPS (Table 1). Clinicians should take more concern regarding sexual dysfunction and pain in IC/BPS patients, and more randomized controlled studies should be conducted to better understand correlations and diagnostic differentiations.

**Table 1:** Characteristics of FSD observed in women with IC/BPS

	Age	Types of FSD	Prevalence (%)	Other associated conditions
Verit et al. [8]^a^	34.73 ± 8.07 years	NA	67.8 (78 of 112)	NA
Gardella et al. [28]	38.7 ± 12 years	Vulvodynia Dyspareunia	Vulvodynis: 85.1 (40 of 47)	LUTS (frequency, urgency)
			Vaginal burning: 65.9 (31 of 47)	
			Dyspareunia: 31.9 (15 of 47)	
Carrico et al. [24]	52 years (range, 19–90)	Vulvodynia	Vulvar pain: 48.1 (91/189)	Sexually transmitted infections (genital warts, positive HPV, gonorrhea, chlamydia)
			Pain with intercourse: 66.9 (117/175)	
Yoon et al. [9]	51.0 ± 14.7 years	Vulvodynia Dyspareunia	NA	LUTS (frequency, urgency)
Bogart et al. [27]	43.6 ± 16.7	NA	Women experienced with sexual dysfunction symptoms: 88 (866/985)	NA
	years			
Gardella et al. [23]	38.2 ± 11.3 years	Vulvodynia Dyspareunia	Spontaneous vulvodynia: 23.4 (11 of 47)	NA
			Provoked vulvodynia: 74.5 (35/47)	
			Localized vulvodynia: 80.9 (38/47)	
			Generalized vulvodynia: 17 (8/47)	
			Dyspareunia: 87.2 (41/47)	

^a^This study was done in the women diagnosed of chronic pelvic pain. *FSD* female sexual dysfunction, *IC/BPS* interstitial cystitis/bladder pain syndrome, *LUTS* lower urinary tract symptom, *NA* Not applicable

## Conclusions

Both vulvodynia and IC/BPS are common and irritable conditions that disrupt normal life and reduce QoL in women. Vulvodynia also manifests itself along with IC/BPS and there are several reports supporting an association between the two diseases. Unfortunately, knowledge concerning vulvodynia and IC/BPS is inadequate when considering the clinical impact and importance of these two conditions. Therefore, there is an essential need for further studies that delve into discovering the features of vulvodynia and IC/BPS.

## Data Availability

Not applicable.

## Abbreviations

CPP: Chronic pelvic pain
CSS: Central sensitivity syndrome
FSD: Female sexual dysfunction
IC/BPS: Interstitial cystitis/bladder pain syndrome
ICSM: International consultation on sexual medicine
LUTS: Lower urinary tract symptoms
OAB: Overactive bladder
QoL: Quality of life
TRPV1: Transient receptor potential V1
